# Behandlungssituation der Fournier-Gangrän an deutschen Universitätskliniken – eine nationale Umfrage zur Deskription aktueller Standards und resultierende Implikationen zur Planung einer Registerstudie

**DOI:** 10.1007/s00120-021-01461-4

**Published:** 2021-02-09

**Authors:** Jennifer Kranz, Florian M. E. Wagenlehner, Joachim Steffens, Oliver W. Hakenberg, Laila Schneidewind

**Affiliations:** 1 Klinik für Urologie und Kinderurologie, St.-Antonius Hospital gGmbH, Eschweiler, Deutschland; 2 Universitätsklinik und Poliklinik für Urologie, Universitätsklinikum Halle (Saale), Halle (Saale), Deutschland; 3 Klinik für Urologie, Kinderurologie und Andrologie, Justus-Liebig-Universität-Gießen, Gießen, Deutschland; 4 Urologische Klinik und Poliklinik, Universitätsmedizin Rostock, Schillingallee 35, 18055 Rostock, Deutschland

**Keywords:** Harnwegsinfektionen, Sepsis, Fournier-Gangrän, Letalitätsrate, Registerstudie, Urinary tract infections, Sepsis, Fournier’s gangrene, Mortality rate, Registry study

## Abstract

**Hintergrund:**

Die Fournier-Gangrän (FG) ist ein seltenes, aber lebensbedrohliches Krankheitsbild, dessen Prognose in den letzten Jahren nicht wesentlich verbessert werden konnte.

**Ziel der Arbeit:**

Primäres Studienziel ist die Deskription der Therapiesituation von FG an deutschen Universitätskliniken. Als sekundäres Studienziel sollen Faktoren identifiziert werden, die mit einer erhöhten Letalität assoziiert sind. Außerdem sollen die Daten zur Planung einer Registerstudie dienen.

**Material und Methoden:**

Es wurde ein Fragebogen mit 29 Fragen an die Mitglieder des Verbands Universitätsklinika Deutschlands sowie eine 3‑malige Erinnerung zur Studienteilnahme im Zeitraum April bis Juni 2020 versandt. Die Datenverwaltung und statistische Analyse erfolgte mit SPSS 26.0 (SPSS Inc., Chicago, Il, USA).

**Ergebnisse:**

Die Antwortrate betrug 88,9 %. Im Median werden jährlich 5 Patienten mit einem medianen Alter von 60 Jahren an deutschen Universitätsklinika mit der Diagnose FG therapiert. Insgesamt stellt sich die Therapiesituation sehr heterogen dar, insbesondere hinsichtlich der empirischen Erstantibiose. Ein signifikanter Risikofaktor für eine Letalität > 20 % konnte identifiziert werden: Intensivaufenthalt von größer gleich 10 Tagen (*p* = 0,039). Die Hälfte (50,0 %) der Befragten geben an, dass sich die Prognose der Erkrankung nicht wesentlich verbessert hat. Weiterhin halten viele der Befragten die Letalität für inakzeptabel hoch, daher befürworten 84,4 % eine Registerstudie. Aus den Kommentaren der Befragten konnten zahlreiche Vorschläge sowie Implikationen für die Planung einer solchen Studie abgeleitet werden, wie z. B. die histologische Sicherung der FG.

**Schlussfolgerung:**

Die Therapiesituation der FG ist heterogen. Allerdings sind die Therapieergebnisse weiterhin inakzeptabel und schwer prognostizierbar.

## Abstract (en)

**Background:**

Fournier’s gangrene (FG) is a sporadic and life-threatening disease, but the outcome has not improved in recent years.

**Objectives:**

The primary aim of this study was the description of current practice patterns in German academic medicine. The secondary aim was the identification of factors associated with a higher mortality rate. Furthermore, the data will be used for the planning of a registry study.

**Materials and methods:**

A 29-item nonvalidated questionnaire was sent to German University Medical Centers (Department of Urology), including three reminders from April through June 2020. Data management and analysis were performed with SPSS 26.0.

**Results:**

The response rate was 88.9%. A median of 5 patients (median age 60.0 years) with FG were treated annually in German University Medical Centers. The contemporary practice patterns are very heterogeneous, especially in terms of empirical antibiotic treatment. Only one significant risk factor for a mortality rate higher than 20.0% was identified—intensive care treatment for ≥10 days (*p* = 0.039). In addition, 50% stated that outcome of FG has not improved in recent years. Furthermore, the majority of the respondents think that mortality is still too high. Consequently, 84.4% support a registry study. From the answers to the open questions we received a variety of suggestions for planning such a study, e.g., histological confirmation of the disease.

**Conclusion:**

Treatment of FG is currently very heterogeneous. Furthermore, treatment outcomes are often unacceptable and difficult to predict.

## Hintergrund und Fragestellung

Die Fournier-Gangrän (FG) ist eine sporadische, lebensbedrohliche, nekrotisierende Infektion des Perineums, der Perinealregion und des äußeren Genitales [1–3]. Da die Inzidenz der Erkrankung sehr niedrig ist, ist auch die Datenlage äußert limitiert und stammt mehrheitlich aus kleinen retrospektiven Fallserien [1, 4–16]. Weiterhin hat die FG eine schlechte Prognose. Frühe Untersuchungen zeigten eine Mortalitätsrate von 20–88 % [1, 2, 17–19], während zwei Studien aus dem Jahr 2017 eine Mortalitätsrate von 25–26 % kalkulierten [3, 12], was vor dem Hintergrund der modernen Medizin eine ernst zu nehmende Situation darstellt. Außerdem berichteten Kranz et al. im Jahr 2018, dass trotz ausgedehnterer intensivmedizinischer Betreuung die Prognose von FG in den letzten 10 Jahren nicht verbessert werden konnte [1].

Schlüsselpunkte für eine erfolgreiche Therapie der FG sind sofortiges radikales chirurgisches Débridement sowie eine Breitspektrumantibiose und ggf. intensivmedizinische Betreuung. Dies stellt seit Jahren den Therapiestandard dar [20]. Inwieweit andere Therapieformen, wie z. B. die hyperbare Oxygenierung (HBO), zur Verbesserung der Therapie und der Prognose der FG beitragen, wird weiter kontrovers diskutiert [21].

Aufgrund dieser Situation mit einer schlechten Prognose sowie einer limitierten, teils widersprüchlichen Datenlage zur Behandlung der FG ist unser primäres Studienziel, mittels einer nationalen Umfrage die Therapiesituation der FG zunächst an deutschen Universitätskliniken darzustellen. Als sekundäres Studienziel sollten Faktoren identifiziert werden, die mit einer erhöhten Letalität assoziiert sind. Abschließend ist das Ziel dieser Arbeit, aus den erhobenen Daten eine nationale Registerstudie für die FG zu planen, da es aufgrund der Rarität der Erkrankung sehr schwierig ist, auch prospektive multizentrische klinische Studien durchzuführen. Die Mortalitätsrate aber weiterhin inakzeptabel hoch ist.

## Studiendesign

Der Fragebogen wurde anhand von Leitlinien zur Umfrageerstellung und Publikation von Studienumfragen entwickelt. Diese sind online über das Portal equator-network.org, einer internationalen Initiative zur Verbesserung von Studienqualität und Berichterstattung, verfügbar [22, 23].

Der erste Schritt in der Fragebogenentwicklung war die zielgerichtete Formulierung der Fragestellungen. Hierfür wurde zunächst eine explorative Literaturrecherche in MEDLINE durch die Artikelautoren und nachfolgend die Fragebogenerstellung durchgeführt.

Der endgültige, nicht-validierte Fragebogen enthält insgesamt 29 Fragen; 13 Fragen bieten die Möglichkeit der offenen Antwort. Die restlichen 16 Fragen sind Multiple Choice, zusätzlich gab es die Möglichkeit, auch bei diesen Antworten einen Kommentar einzufügen.

Nachfolgend wurde der vollständige Fragebogen sowie eine Einladung zur Studienteilnahme per E‑Mail an die urologischen Kliniken des Verbands Universitätsklinika Deutschlands (36 Vollmitglieder, Stand April 2020) versandt. Im Zeitraum April bis Juni 2020 erinnerten die Autoren insgesamt 3‑mal an die Möglichkeit zur Teilnahme. Als Zielpopulation wurden Universitätskliniken gewählt, da es sich bei diesen stets um Maximalversorger handelt.

Bei dieser Umfrage wurden die ethischen Standards der Deklaration von Helsinki von 1964 und der späteren Nachträge eingehalten. Für diesen Studientyp bedarf es keiner separaten Einwilligung.

Nur vollständige Datensätze gingen in die Analyse ein und wurden in das Statistik Programm SPSS 26.0 (SPSS Inc., Chicago, Il, USA) transferiert sowie mit diesem analysiert. Zunächst wurde für nummerische Variablen mittels Kolmogorov-Smirnov-Test ermittelt, ob eine Normalverteilung vorliegt. Deskriptive Statistik wurde unter Angabe von Mittelwert und Standardabweichung (SD) bei normalverteilten Variablen sowie von Median und Interquartilsabstand (IQR) bei nicht-parametrischen Daten durchgeführt. Für kontinuierliche normalverteilte Variablen wurde der t‑Test verwendet bzw. bei normalverteilten kategorialen Variablen der χ^2^-Test bzw. der Fisher-Test. Für nicht-parametrische Variablen (kontinuierlich und kategorial) wurde der Mann-Whitney-U-Test durchgeführt. Alle berichteten *p*-Werte basieren auf einer zweiseitigen Hypothese, wobei *p* < 0,05 wurde dabei als signifikant gewertet.

## Ergebnisse

### Antwortrate und Charakterisierung der therapeutischen Situation

Wir erhielten Antwort von 32 der 36 deutschen Universitätskliniken (88,9 %). Weiterhin wurden alle Fragebögen vollständig ausgefüllt. Die Tab. 1 gibt einen umfassenden Überblick über die aktuelle therapeutische Situation bei der FG. Im Median werden pro Jahr 5 Primärfälle beobachtet. Eine Standardarbeitsanweisung (SOP) zur Therapie der FG benutzen 10 (31,3 %) Kliniken. Wesentliche Punkte der verwendeten SOP sind möglichst rasche Einleitung der Therapie (*n* = 8; 25,0 %), regelhafte intensivmedizinische Betreuung (*n* = 4; 12,5 %), obligater „second look“ (*n* = 2; 6,3 %) sowie eine rasche CT-Bildgebung (*n* = 2; 6,3 %). Hinsichtlich der empirischen Erstantibiose zeigt sich ein sehr heterogenes Bild, am häufigsten wird Piperacillin/Tazobactam (*n* = 10; 31,3 %) verwendet. Es gab eine (3,1 %) Universitätsklinik an, keine empirische Erstantibiose zu verwenden. Lediglich 2 (6,3 %) Universitätsklinika verwenden ein Scoring System für diese Erkrankung, dies ist in beiden Fällen der „Fournier Gangrene Severity Index“ (FGSI). Gelingt ein Erregernachweis, ist dies regelhaft im Wundabstrich. 4‑mal (12,5 %) wurde die Häufung eines bestimmten Erregers beobachtet, namentlich einmal Streptococcus aeruginosa (3,1 %), einmal Anaerobier spp. (3,1 %) sowie 2‑malig Staphylococcus spp. (6,3 %). Ebenfalls wurde 4‑mal (12,5 %) eine Häufung von multiresistenten Erregern dokumentiert, in allen Fällen handelte es sich um multiresistente gramnegative Bakterien (3MRGN). Die Patienten werden nur in 5 (15,6 %) Kliniken selbst nachgesorgt, dabei in 4 (12,5 %) Fällen durch individuelle Schemata in der eigenen urologischen Ambulanz und in einem (3,1 %) Fall durch die Kollegen der plastischen Chirurgie. Daten zur Lebensqualität der FG-Patienten werden kaum erhoben, nur eine (3,1 %) Universitätsklinik verwendet einen Fragebogen zur Beurteilung des subjektiven Wohlbefindens der Patienten. Bezüglich der Nennung wesentlicher Outcome-Faktoren (hier war eine Mehrfachnennung möglich) zeigt sich ein heterogenes Bild, wobei die Zeit bis zur Therapie und die Komorbiditäten des Patienten am häufigsten berichtet worden sind. Bei der Frage, ob sich die Prognose der FG in den letzten Jahren verbessert hat, beantworteten 50 % dies mit ja. Die Befragten, die von einer Prognoseverbesserung ausgehen, sollten nachfolgend die wesentlichen Faktoren, die zu dieser Verbesserung geführt haben nennen (auch Mehrfachnennungen waren möglich). Diese Prognosefaktoren waren: verbesserte intensivmedizinische Betreuung (*n* = 812; 37,5 %), verkürzte Zeit bis zur Therapie (*n* = 88; 25,0 %) sowie die hyperbare Oxygenierung (HBO; *n* = 81; 3,1 %).

**Table 1:** 

Charakteristikum	*n* (%)		Median (IQR)
Primärfälle pro Jahr	–		5,0 (3,0–7,7)
SOP vorhanden	Ja	10 (31,3)	–
	Nein	22 (68,7)	
Empirische Antibiose	Piperacillin/Tazobactam	10 (31,3)	–
	Piperacillin/Tazobactam plus Vancomycin oder Metronidazol oder Clindamycin	7 (21,9)	
	Carbapenem plus Vancomycin oder Metronidazol oder Clindamycin	5 (15,6)	
	Carbapenem	5 (15,6)	
	Ampicillin/Sulbactam plus Flucloxacillin	1 (3,1)	
	Gentamicin	1 (3,1)	
	Carbapenem plus Linezolid	1 (3,1)	
	Clindamycin	1 (3,1)	
	Keine 1 (3,1)	1 (3,1)	
HBO angewandt	Ja	4 (12,5)	–
	Nein	28 (87,5)	
Stunden bis zur OP	–		2,0 (1,0–4,0)
Stunden bis zur Antibiose	–		0,5 (0,5–1,0)
Verwendung von Scoring-System	Ja	2 (6,3)	–
	Nein	30 (93,7)	
Regelhafter Erregernachweis	Ja	24 (75,0)	–
	Nein	8 (25,0)	
Häufung bestimmter Erreger	Ja	4 (12,5)	–
	Nein	28 (87,5)	
Zunahme multiresistenter Erreger	Ja	4 (12,5)	–
	Nein	28 (87,5)	
Anzahl Wunddébridements	–		4,0 (3,0–5,0)
Art der Harnableitung	Suprapubischer Katheter	23 (71,9)	–
	Transurethraler Katheter	5 (15,6)	
	Ileumconduit	4 (12,5)	
Zusammenarbeit mit plastischer Chirurgie	Ja	26 (81,3)	–
	Nein	6 (18,7)	
Tage ITS-Aufenthalt	–		7,0 (5,0–10,0)
Tage stationärer Aufenthalt	–		21,0 (20,0–28,0)
Nachsorge in Klinik	Ja	5 (15,6)	–
	Nein	27 (87,4)	
Lebensqualitätsdaten erhoben	Ja	1 (3,1)	–
	Nein	31 (96,9)	
Letalitätsrate (%)	–		15,0 (15,0–30,0)
Wesentlicher Outcome-Faktor	Zeit bis zur Therapie	19 (59,4)	–
	Komorbiditäten	15 (46,9)	
	Radikales Débridement	8 (25,0)	
	Ausmaß des Befunds	4 (12,5)	
	Allgemeinzustand	3 (9,4)	
	Diabetes mellitus	2 (6,3)	
	Adipositas	1 (3,1)	
	Alter	1 (3,1)	
	Sepsisparameter	1 (3,1)	
	ITS-Aufenthalt	1 (3,1)	
	Immunsuppression	1 (3,1)	
Wesentliche Prognoseverbesserung	Ja	16 (50,0)	–
	Nein	16 (50,0)	
Registerstudie sinnvoll	Ja	27 (84,4)	–
	Nein	5 (15,6)	

*SOP* Standardarbeitsanweisung, *HBO* hyperbare Oxygenierung, *OP* Operation, *ITS* Intensivstation, *IQR* Interquartilsabstand

### Demographische Charakterisierung der Patientenpopulation

Das mediane Patientenalter der an FG Erkrankten beträgt im Median 60,0 Jahre. Tab. 2 gibt einen Überblick über die demographischen Charakteristika der FG-Patienten an den deutschen Universitätsklinika. Hinsichtlich der Komorbiditäten war es möglich mehrere zu nennen, dabei sind die häufigsten Komorbiditäten Diabetes mellitus (*n* = 27; 84,4 %) und Adipositas (*n* = 10; 31,3 %).

**Table 2:** 

Charakteristikum	*n* (%)		Median (IQR)	Kommentar
Patientenalter	–		60,0 (56,3–60,0)	–
Komorbiditäten	Diabetes mellitus	27 (84,4)	–	–
	Adipositas	10 (31,3)		
	KHK	4 (12,5)		
	Nikotinabusus	1 (3,1)		
	Immunsuppression	1 (3,1)		
	DK-Dauerversorgung	1 (3,1)		
	Schwere Sepsis	1 (3,1)		
Zusammenhang mit oralen Antidiabetika	Ja	2 (6,3)	–	Metformin 1 (3,1) Gliflozine 1 (3,1)
	Nein	30 (93,7)		

*KHK* koronare Herzkrankheit, *DK* Dauerkatheter, *IQR* Interquartilsabstand

### Assoziationen mit einer Letalitätsrate >20 %

Als sekundäres Studienziel testen wir auf Faktoren, die evtl. mit einer angegebenen Letalitätsrate >20 % assoziiert sind. Diese hohe Rate wurde von insgesamt 15 (45,9 %) Universitätskliniken berichtet. Die Tab. 3 gibt einen Überblick über die entsprechenden Testergebnisse. Interessanterweise ist lediglich ein Aufenthalt auf der Intensivstation von ≥10 Tagen mit einer erhöhten Letalität statisch signifikant assoziiert (*p* = 0,039).

**Table 3:** 

Charakteristikum	*n* (%)	*p*-Wert
Verwendung von HBO	4 (12,5)	0,356
Kein Erregernachweis	8 (25,0)	0,840
Zunahme multiresistenter Erreger	4 (12,5)	0,895
Harnableitung SPK	23 (71,9)	0,536
Harnableitung DK	5 (15,6)	0,867
Harnableitung Ileumconduit	4 (12,5)	0,529
Empirische Einzelantibiose	17 (53,1)	0,156
Stunden bis zur Operation >3	12 (37,5)	0,787
Stunden bis zur Operation >4	3 (9,4)	0,478
Stunden bis zur Antibiose >2	1 (3,1)	0,287
Anzahl Wunddébridements >3	18 (56,3)	0,758
ITS-Aufenthalt größer 5 Tage	20 (62,5)	0,242
ITS-Aufenthalt größer gleich 10 Tage	*13 (40,6)*	*0,039*

*HBO* hyperbare Oxygenierung, *SPK* suprabubischer Katheter, *DK* transurethraler Katheter, *ITS* Intensivstation

### Wichtige Kommentare und Anregungen zur Planung einer deutschen Registerstudie

Die Mehrheit der Befragten (*n* = 27; 84,4 %) befürworten die Etablierung einer deutschen Registerstudie für das seltene Krankheitsbild FG oder halten diese sogar für sehr sinnvoll, insbesondere vor dem Hintergrund, dass die Letalitätsrate weiterhin inakzeptabel hoch sei. Fünf (15,6 %) der Befragten lehnen dieses Vorgehen allerdings ab und nennen als Gründe: Die Fälle seien zu heterogen, es sei keine Beteiligung zu erwarten, es gebe wichtigere Erkrankungen und bei diesen seien die finanziellen Mittel sinnvoller eingesetzt, es sei zu viel Aufwand und ein Befragter machte keine Angabe zu den Gründen. Die Vorteile einer solchen Registerstudie seien laut den Befragten die Möglichkeit, deutschlandweite Standards zur Therapie zu etablieren, Zentren mit optimaler intensivmedizinischer Betreuung oder der Möglichkeit zu HBO zu schaffen, ein Scoring-System für alle zu entwickeln und damit zu definieren, welche FG-Patienten tatsächlich in einem Zentrum therapiert werden müssen sowie die Vorrausetzungen und den Nutzen eines vakuumassistierten (VAC) Wundverschlusses zu definieren. Zusätzlich wurden von einigen Befragten konkrete Vorschläge für die Registerstudie gemacht, so sollte im Rahmen der Studie auf eine histologische Sicherung der FG bestanden sowie ein Erregernachweis aus dem Gewebe angestrebt werden. Die Bestimmung von Procalcitonin (PCT) und Laktatdehydrogenase (LDH) aus dem Serum sollte obligat sein und die Fachgebiete Chirurgie als auch Gynäkologie sollten in die Studie mit einbezogen werden, da diese Kollegen häufiger weibliche Patienten mit FG behandeln und weibliche Patienten so übersehen werden würden. Ein Befragter machte konkrete Vorschläge für weitere Datenerhebung innerhalb einer solchen Registerstudie, diese sind in Tab. 4 dargestellt.

**Table 4:** 

Parameter/Frage
FG der Frau miterheben/dafür muss Chirurgie und Gynäkologie mit einbezogen werden
Wurde eine unilaterale oder eine bilaterale Orchiektomie durchgeführt?
Wie oft wird die simultane Anus-praeter-Anlage durchgeführt?
Wie oft ist der Perinealbereich mitbetroffen/genaues Ausmaß der Erkrankung

*FG* Fournier-Gangrän

## Diskussion

Wir führten eine Umfrage zur Therapiesituation der FG an deutschen Universitätskliniken durch. Unseres Wissens handelt es sich um die erste Studie dieser Art, daher ergibt sich die Chance, aus den gewonnenen Daten eine prospektive, deutsche Registerstudie zu planen. Dies ist das erklärte Ziel dieser Untersuchung.

Insgesamt stellt sich die Therapie der FG an deutschen Universitätskliniken sehr heterogen dar, insbesondere hinsichtlich der empirischen Antibiose. Erfreulicherweise beträgt die Letalitätsrate in dieser Untersuchung im Median 15 %, dies liegt unter den Raten, die in der Literatur beschrieben werden [1–3, 12, 17–19]. Weiterhin ist erfreulich, dass keine erhebliche Zunahme von multiresistenten Erregern beobachtet worden ist, dies stimmt mit den Daten aus der Literatur überein [1]. Trotzdem kommentiert die Mehrheit der Befragten, dass die Letalitätsrate und die Prognose der Erkrankung inakzeptabel hoch sei und 50 % der Befragten berichten, dass es keine Verbesserung der Prognose in den letzten Jahren gegeben hat. Dies sind die wesentlichen Gründe dafür, dass die Mehrheit der Beteiligten eine Registerstudie befürwortet.

Im Gegensatz dazu zeigt sich bzgl. des Patientenkollektives, der Komorbiditäten und der Prognoseparameter ein homogenes Bild, so ist z. B. Diabetes mellitus auch in der klinischen Praxis die häufigste Begleiterkrankung. Allerdings scheint es nicht zu gelingen, den Verlauf der Erkrankung gut vorherzusagen, so konnten wir lediglich einen Faktor identifizieren, der signifikant mit einer Letalitätsrate >20 % assoziiert ist. Dies ist ein ITS Aufenthalt von ≥10 Tagen und erscheint wenig überraschend, da morbidere Patienten eben mehr intensivmedizinische Betreuung benötigen. Daraus lässt sich konsequenterweise schlussfolgern, dass ein schwerer bzw. letaler Verlauf der FG nicht gut prognostizierbar ist. Hieraus wiederum ergeben sich Implikationen für eine Registerstudie, so könnten Scores bzw. Modellsysteme entwickelt werden, um Patienten zu identifizieren, die von bestimmten Therapieformen profitieren. Wer muss in einem Zentrum mit entsprechender intensivmedizinischer Expertise behandelt werden? Wer profitiert von der HBO? Wer benötigt wie und wann eine VAC-Therapie? Wie sieht der rekonstruktive Aspekt der Defektdeckung nach überstandener Infektion aus? Dies sind Fragen, die zum einen nicht abschließend geklärt sind und zum anderen durch eine solche Registerstudie beantwortbar werden würden. Selbst bei einigen Kommentaren der Befragten wurden genau diese Fragen aufgeworfen, was deren Dringlichkeit unterstreicht.

Dankenswerterweise wurden bereits sogar konkrete Vorschläge von den Befragten zur Planung einer solchen Studie gemacht. Diese sind äußerst vielfältig und wichtig, dabei reichen diese von der Bestimmung der LDH bis hin zur Einbeziehung der Fachgebiete Chirurgie sowie Gynäkologie. Letzteres ist ein essentieller Vorschlag, da sonst die FG der Frau nur unzureichend abgebildet werden würde. Weiterhin haben wir aus dieser Erhebung gelernt, dass einige Punkte zu wenig dokumentiert werden und zukünftig mit erhoben werden sollten, z. B. die Lebensqualität. Diese ist von erheblicher Bedeutung für den Patienten, findet aber bei der FG scheinbar kaum Beachtung, daher sind wir der Überzeugung, dass innerhalb einer Registerstudie Daten zu Lebensqualität bzw. „patient reported outcomes“ mit erhoben werden sollten.

Selbstverständlich gab es auch kritische Stimmen zu einer Registerstudie. Für diese kritischen Diskussionen und Anmerkungen sind wir sehr dankbar. Es entspricht einer Tatsache, dass eine Registerstudie für solch eine seltene Erkrankung einen erheblichen Aufwand inklusive finanzieller Ressourcen darstellt. Allerdings sollte dies für uns Ärzte aus ethischer Sicht kein Argument sein, insbesondere vor dem Hintergrund, dass Forschung zu seltenen Erkrankungen kaum oder nur unzureichend betrieben wird [24]. Die Prognose der FG ist im Jahr 2020 weiterhin schlecht, daher sollte an einer Verbesserung der Therapie für diese Patienten gearbeitet werden. Außerdem entspricht es einer Tatsache, dass sich die Situation sehr heterogen darstellt. Doch eine Registerstudie bietet die Möglichkeit z. B. durch Modellsysteme Muster zu erkennen – vielleicht sogar eher als eine prospektive klinische Multicenterstudie, die aufgrund der Seltenheit von FG auch schwierig durchzuführen sein würde.

Natürlich weist diese Umfrage einige Limitationen auf, wie den nicht-validierten Fragebogen oder die zahlreichen offenen Antwortmöglichkeiten. Allerdings wollten wir mit dieser Studie auch ein möglichst offenes und umfassendes Bild erhalten, um eben weitere Untersuchungen zur FG sinnvoll planen zu können.

Zusammenfassend stellt sich die Therapiesituation der FG an deutschen Universitätskliniken sehr heterogen dar. Es handelt sich weiterhin um eine schwere seltene Erkrankung, die schwer prognostizierbar und deren Letalität immer noch zu hoch ist. Eine nationale Registerstudie ist ein möglicher sinnvoller Forschungsansatz, um die Behandlungssituation zu verbessern. Aus dieser Umfrage ergeben sich wichtige Implikationen zur Planung einer solchen Studie, wie z. B. die Entwicklung von Modellsystemen, um vorhersagen zu können, wer von bestimmten Therapieformen profitiert.

## Fazit für die Praxis

- Die Fournier-Gangrän (FG) ist ein seltenes Krankheitsbild, dessen Therapiesituation sich in Deutschland sehr heterogen darstellt.
- Therapieergebnisse der FG sind weiterhin nicht zufriedenstellend.
- Der Verlauf der FG ist schwer prognostizierbar.
- Eine nationale Registerstudie FG bietet eine verhältnismäßig einfache Möglichkeit, zahlreiche offene Fragen zur Therapie der FG zu beantworten.
